# Supplementary material for: Ubiquitin B, Ubiquitin C, and β-Catenin as Promising Diagnostic and Prognostic Tools in Prostate Cancer
Source: Cancers (Basel). 2024 Feb 23;16(5):902. doi: 10.3390/cancers16050902 (PMC10930646; doi:10.3390/cancers16050902)
Supplement: Supplementary file 1 [file cancers-16-00902-s001.zip › cancers-2847046-supplementary.pdf]

Table S1. A detailed clinical characterization of patients within the institutional cohort.

Variables	n=67
**Age (years)**	
**⩽65**	32 (47.76)
**>65**	35 (52.24)
**Gleason score**	
**GS 6**	3 (4.48)
**GS 7**	35 (52.24)
**GS 8**	11 (16.42)
**GS 9**	18 (26.87)
**Grade group**	
**group 1**	3 (4.48)
**group 2**	11 (16.42)
**group 3**	24 (35.82)
**group 4**	11 (16.42)
**group 5**	18 (26.87)
**pT status**	
**T2**	9 (13.43)
**T3-T4**	58 (86.57)
**pN status**	
**Nx**	1
**N0**	43 (65.15)
**N1**	23 (34.85)
**PSA**	
**x**	2
**⩽10 ng/ml**	27 (41.54)
**>10 ng/ml**	38 (58.46)

Table S2. A detailed clinical characterization of patients within the TCGA cohort.

Variables	n = 495
**Age (years)**	
**≤ 60**	222 (44.85)
**> 60**	273 (55.15)
**Gleason score**	
**GS 6**	45 (9.09)
**GS 7**	247 (49.90)
**GS 8**	64 (12.93)
**GS 9**	135 (27.27)
**GS 10**	4 (0.81)
**pT status**	
**x**	7
**T2**	187 (38.32)
**T3-T4**	301 (61.68)
**pN status**	
**x**	73
**N0**	342 (81.04)
**N1**	80 (18.96)
**PSA**	
**x**	56
**<4 ng/ml**	368 (83.83)
**>4 ng/ml**	71 (16.17)

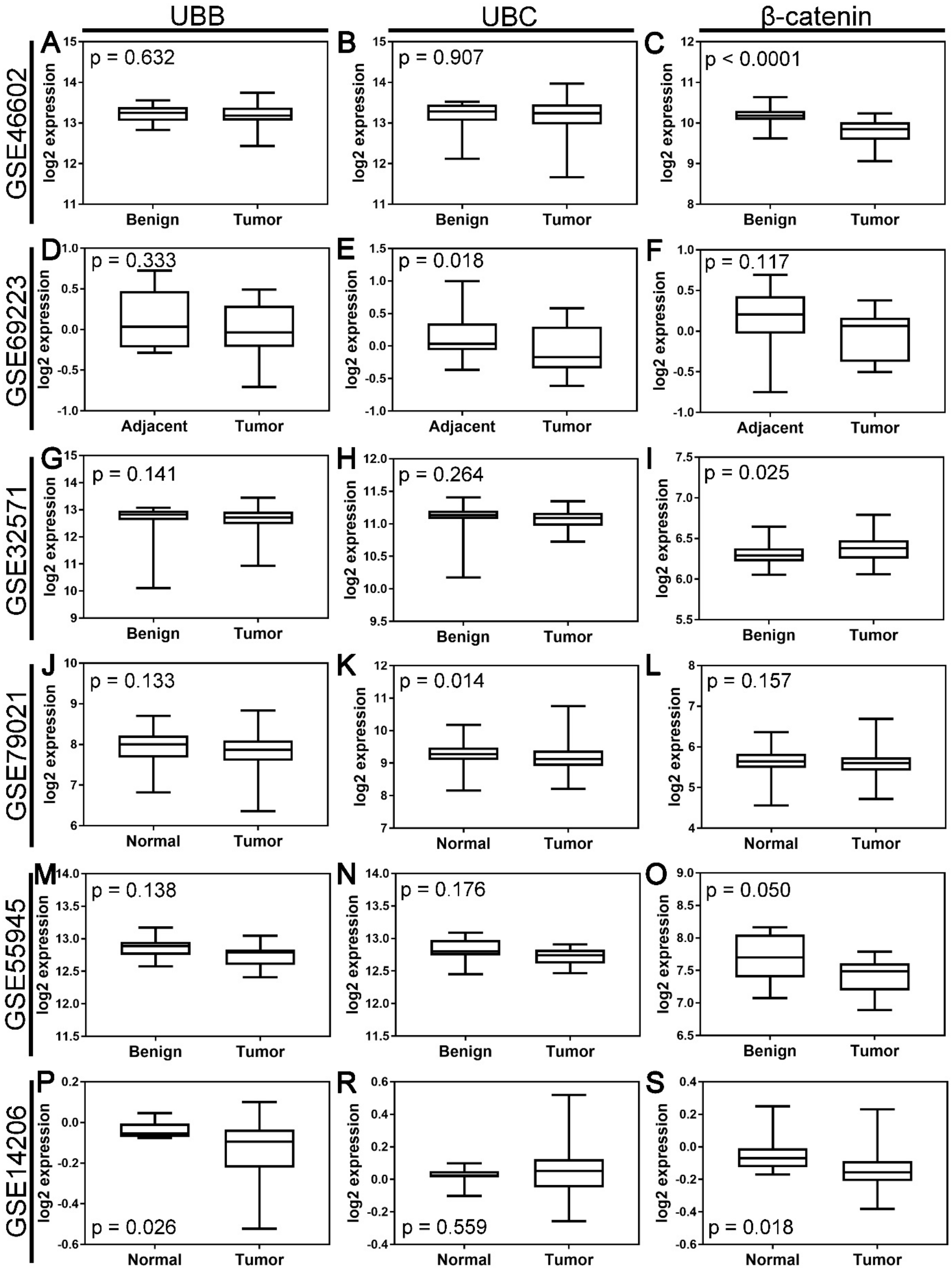

Figure S1. Expression of *UBB, UBC* and *CNTTB1* in prostate cancer compared to non-cancerous control tissue based on the gene chip data derived from the Gene Expression Omnibus (GEO) database through the ShinyGEO web-based tool. Boxplot graphs of (A) UBB, (B) UBC and (C) *CNTTB1 (*β-Catenin) expression levels in tumor tissue (n = 36) and benign prostate glands (n = 14); (D) *UBB,* (E) *UBC* and (F) *CNTTB1 (*β-Catenin) expression levels in tumor tissue (n = 15) and matched adjacent normal tissue (n = 15); (G) *UBB,* (H) *UBC* and (I) *CNTTB1 (*β-Catenin) expression levels in tumor tissue (n = 59) and matched benign tissue sample (n = 39); (J) *UBB,* (K) *UBC* and (L) *CNTTB1 (*β-Catenin) expression levels in tumor tissue (n = 153) and normal prostate tissue sample (n = 49); (M) *UBB,* (N) *UBC* and (O) *CNTTB1 (*β-Catenin) expression levels in tumor tissue (n = 13) and benign prostate glands (n = 8); (P) *UBB,* (R) *UBC* and (S) *CNTTB1 (*β-Catenin) expression levels in tumor tissue (n = 53) and benign prostate glands (n = 14). The GSE numbers are shown.

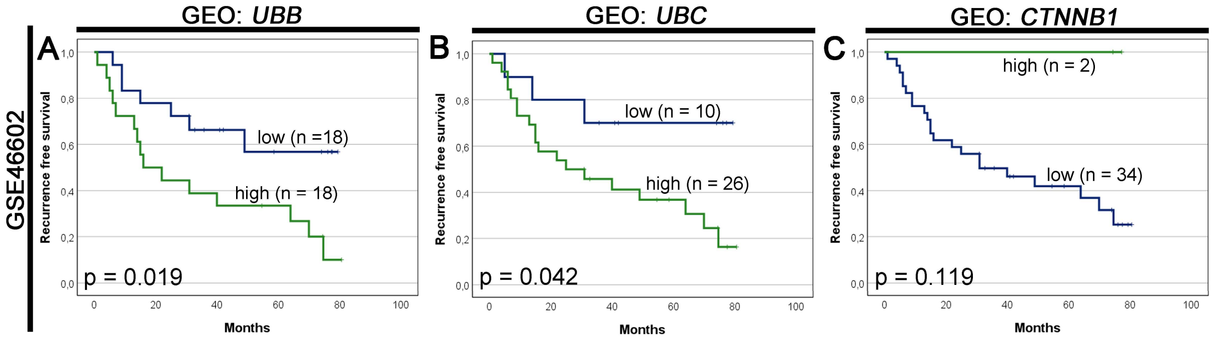

Figure S2. Kaplan-Meier survival curves stratified by expression of (A) *UBB*, (B) *UBC*, and (C) *CTNNB1* (β-Catenin) in prostate cancer. Microarray-based mRNA expression in publicly available GSE46602 dataset derived from the Gene Expression Omnibus (GEO) database through the ShinyGEO web-based tool.The number of cases (n) for low and high-expression groups is displayed.
